# Physosmotic Induction of Chondrogenic Maturation Is TGF-β Dependent and Enhanced by Calcineurin Inhibitor FK506

**DOI:** 10.3390/ijms23095110

**Published:** 2022-05-04

**Authors:** Holger Jahr, Anna E. van der Windt, Ufuk Tan Timur, Esther B. Baart, Wei-Shiung Lian, Bernd Rolauffs, Feng-Sheng Wang, Thomas Pufe

**Affiliations:** 1 Department of Anatomy and Cell Biology, University Hospital RWTH Aachen University, 52074 Aachen, Germany; u.timur@maastrichtuniversity.nl (U.T.T.); tpufe@ukaachen.de (T.P.); 2 Department of Orthopaedic Surgery, Maastricht University Medical Center, 6229 HX Maastricht, The Netherlands; 3 Department of Orthopaedics, Erasmus MC University Medical Center, 3015 GD Rotterdam, The Netherlands; a.vanderwindt@erasmusmc.nl; 4 Department of Obstetrics & Gynaecology, Erasmus University Medical Center, 3015 GD Rotterdam, The Netherlands; e.baart@erasmusmc.nl; 5 Core Laboratory for Phenomics and Diagnostics, Department of Medical Research, College of Medicine, Chang Gung University, Kaohsiung Chang Gung Memorial Hospital, Kaohsiung 83301, Taiwan; lianws@gmail.com (W.-S.L.); wangfs@ms33.hinet.net (F.-S.W.); 6 Center for Mitochondrial Research and Medicine, Kaohsiung Chang Gung Memorial Hospital, Kaohsiung 83301, Taiwan; 7 G.E.R.N. Research Center for Tissue Replacement, Regeneration & Neogenesis, Department of Orthopedics and Trauma Surgery, Faculty of Medicine, Medical Center, Albert-Ludwigs-University, 79085 Freiburg, Germany; bernd.rolauffs@uniklinik-freiburg.de

**Keywords:** hyperosmolarity, chondrocyte, differentiation, ATDC5, FK506, calcineurin, minor collagens, TGFBI, BMP signaling, FKBP-12

## Abstract

Increasing extracellular osmolarity 100 mOsm/kg above plasma level to the physiological levels for cartilage induces chondrogenic marker expression and the differentiation of chondroprogenitor cells. The calcineurin inhibitor FK506 has been reported to modulate the hypertrophic differentiation of primary chondrocytes under such conditions, but the molecular mechanism has remained unclear. We aimed at clarifying its role. Chondrocyte cell lines and primary cells were cultured under plasma osmolarity and chondrocyte-specific in situ osmolarity (+100 mOsm, physosmolarity) was increased to compare the activation of nuclear factor of activated T-cells 5 (NFAT5). The effects of osmolarity and FK506 on calcineurin activity, cell proliferation, extracellular matrix quality, and BMP- and TGF-β signaling were analyzed using biochemical, gene, and protein expression, as well as reporter and bio-assays. NFAT5 translocation was similar in chondrocyte cell lines and primary cells. High supraphysiological osmolarity compromised cell proliferation, while physosmolarity or FK506 did not, but in combination increased proteoglycan and collagen expression in chondrocytes in vitro and in situ. The expression of the TGF-β-inducible protein TGFBI, as well as chondrogenic (*SOX9*, *Col2)* and terminal differentiation markers (e.g., *Col10*) were affected by osmolarity. Particularly, the expression of minor collagens (e.g., *Col9*, *Col11*) was affected. The inhibition of the FK506-binding protein suggests modulation at the TGF-β receptor level, rather than calcineurin-mediated signaling, as a cause. Physiological osmolarity promotes terminal chondrogenic differentiation of progenitor cells through the sensitization of the TGF-β superfamily signaling at the type I receptor. While hyperosmolarity alone facilitates TGF-β superfamily signaling, FK506 further enhances signaling by releasing the FKBP12 break from the type I receptor to improve collagenous marker expression. Our results help explain earlier findings and potentially benefit future cell-based cartilage repair strategies.

## 1. Introduction

Articular cartilage is the highly specialized connective tissue of diarthrodial joints and composed of a dense extracellular matrix (ECM) with a sparse distribution of highly specialized chondrocytes. Principal ECM components are water, collagens, and proteoglycans, which together are critical to maintain the tissue’s unique mechanical properties [1]. While osteoarthritis (OA) is now considered a disease of the whole joint, articular cartilage breakdown remains one of its hallmarks [2]. Several signaling pathways contribute to articular cartilage homeostasis, its focal deterioration, and eventually OA progression, such as transforming growth factor beta (Tgf-β) and nuclear factor of activated T cells (NFAT) signaling [3,4].

NFATs are a family of highly regulated transcription factors that have the potential to link many extracellular signals to the nuclear transcriptional machinery [5]. Canonically, the phosphatase calcineurin (Cn) is induced by calcium signaling to subsequently activate the transcription of nuclear factors of activated T cells (NFATs), comprising NFAT1 to NFAT4 (i.e., NFATc1-c4), in different cell types [6], which regulate, amongst others, chondrogenesis [3,7,8]. We recently showed that Cn inhibitors promote the chondrogenesis of human adult osteoarthritic chondrocytes in vitro through stimulating TGF-β1 synthesis [9].Cn-NFAT signaling further receives much attention as targets for the immunosuppressive drugs cyclosporin A (CsA) and FK506 (Tacrolimus) [10]. Both bind to two different intracellular proteins, FK506 to FKBPs and CsA to cyclophilin, respectively, but both inhibit Cn activity and the Cn-mediated dephosphorylation of NFAT1–4 [6,7]. Interestingly, the fifth family member (NFAT5), also known as TonEBP, is not known to be directly regulated by calcium signaling. Instead, NFAT5 is quite uniquely activated by hyperosmotic stress in response to elevated concentrations of extracellular sodium ions [11]. In mammals, NFAT5 dimerization in response to osmotic stress is particularly important in load-bearing connective tissues, such as the intervertebral disc (IVD) [12,13], to protect vertebrae in the spine from impaction. In articular cartilage, which protects bones in articulating joints in a similar way, chondrocytes are also known to be mechano-responsive and react to cartilage deformation, consequently changing extracellular ionic composition [14,15].

Osmolarity is a major biophysical regulator of chondrocyte function; increasing culture medium osmolarity from plasma level to physosmotic values (i.e., ≥ 350 ≤ 480 mOsm) not only improves chondrocyte-specific marker gene expression in vitro for primary cell-based regenerative approaches [16,17], but also shows chondroprotective effects in situ [18] with implications for arthroscopic irrigating solutions [19,20]. We recently showed that NFAT5 is not only important for maintaining and preserving the chondrocyte phenotype, but it also plays a crucial role during the chondrogenic differentiation of different types of stem cells from different species [21]. In particular, ATDC5 cells turned out to be an excellent model that synchronously displays the multistep differentiation process of mesenchymal progenitor cells until hypertrophic differentiation and matrix mineralization [1,2]. Increasing the osmolarity of the differentiation medium with 100 mOsm from a plasma level baseline significantly increased chondrogenic marker expression, such as glycosaminoglycans (GAG) and collagens types II (Col2a1) and X (Col10a1) during the chondrogenic differentiation of these murine chondroprogenitor cells [21].

In normal articular cartilage, tissue fluid represents 65 to 80% of its total weight [22], with collagens and proteoglycans accounting for the remaining dry weight. Collagens are the most abundant structural ECM macromolecules, making up about 60% of the tissue’s dry weight, with collagen type II representing 90 to 95% of all ECM collagen. Consequently, collagen types I, IV, V, VI, IX, and XI are only contributing a minor mass proportion, but help to form and stabilize the collagen type II fibril network. All collagens comprise triple helical α-chains, which help to stabilize the ECM and provide articular cartilage with important shear and tensile properties [1,23,24]. Proteoglycans (PGs) are heavily glycosylated proteins and represent the second-largest group of ECM macromolecules. Articular cartilage contains a variety of functionally essential PGs, including aggrecan, the largest and most abundant by weight [1], which interacts with hyaluronan to form large PG aggregates via link proteins [25].

In mammals, the Tgf-β superfamily of secreted factors comprises three prototypic Tgf-β isoforms, bone morphogenetic proteins (Bmps), Activins, Nodals, and GDFs [26]. TGF-β superfamily signaling is known to govern diverse physiological and pathological processes, such as tissue homeostasis and differentiation, in a wide variety of cell types [27,28]. In cartilage, the TGF-β superfamily regulates, amongst others, chondrocyte differentiation, and in particular the expression of collagens and proteoglycans [29,30,31,32]. The binding of a TGF-β superfamily ligand to its cell surface receptor triggers several signaling cascades, including the one most extensively studied, the Tgf-β/SMAD pathway. Chondrocyte maturation is tightly regulated through the SMAD-mediated control of transcription factor RUNX2 [30,33,34]. TGF-β superfamily receptors have intrinsic serine/threonine kinase activity and the binding of Tgf-β type II receptor (TβRII) causes the phosphorylation of the type I receptor (TβRI), which subsequently activates Tgf-β-specific SMADs. Type I receptors of the TGF-β superfamily members are also known as activin receptor-like kinases (ALKs). ALK5 predominantly transduces Tgf-β signaling, while ALK1 tends to phosphorylate SMAD1/5/8 to activate chondrocyte-specific target gene expression [35,36].

Surprisingly, FK506 further enhanced osmotic stress-induced anabolic ECM markers in human chondrocytes in vitro [16,17] and protected the collagenous ECM in vivo [37]. In vitro, the expression of BMP and TGF-β isoforms as well as TGF-β bioactivity is modulated by osmolarity [9,38], confirming in silico predictions of TGF-β signaling in post-transcriptional responses to osmotic changes in chondrocytes [39].

Given the different intracellular binding partners of FK506 and CsA, we aimed to shed light on how FK506 enhances chondrogenic differentiation under physosmotic conditions. We showed that safe concentrations of 620 nM or less of FK506 under physosmotic culture conditions not only improved proteoglycan and type II collagen content in chondrocytes, but also upregulated minor collagens important for EC maturation, such as type IX and XI. In conclusion, we discovered evidence that FK506 may not exert its effects primarily through the inhibition of Cn, but rather through sequestering FKBP12 away from type I serine/threonine kinase receptors of the TGF-β superfamily.

## 2. Results

### 2.1. Extracellular Physiological Osmolarity Dose-Dependently Influences NFAT5 Nuclear Translocation and Cell Proliferation

**Figure 1:**
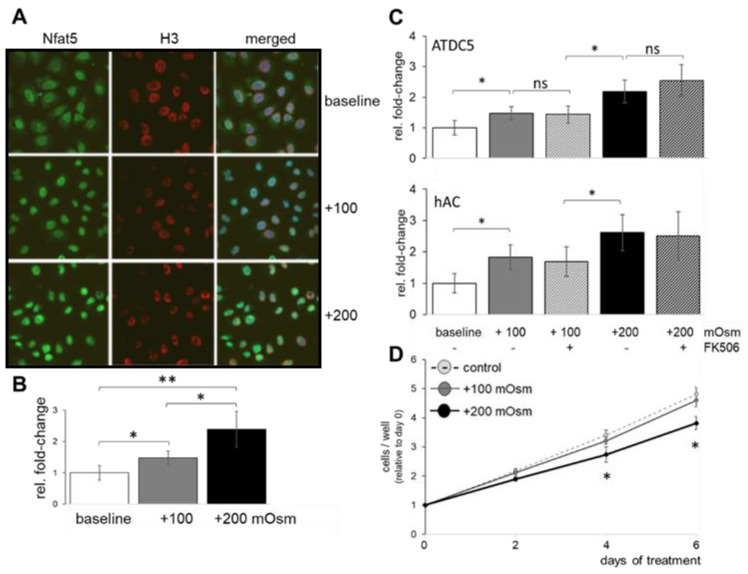
Effects of osmolarity and FK506 on sub-cellular localization of NFAT5 and cell proliferation. (**A**) Representative confocal microscopy images of the sub-cellular localization of NFAT5 (green) and (**B**) quantification of the changes in nuclear-to-cytoplasmic distribution of NFAT5 in ATDC5 cells within 2 h. (**C**) Increasing culture medium osmolarity over plasma level (baseline) by +100 and +200 mOsm/kg, respectively, significantly changed sub-cellular distribution of NFAT5 in ATDC5 cells and human articular chondrocytes (hAC), while adding FK506 (620 nM) did not. (**D**) Increasing the osmotic value of the culture medium beyond +100 mOsm/kg negatively influenced ATDC5 cell proliferation. Ubiquitously expressed histone H3 (H3), red; ns, not significant; *, *p* < 0.05; **, *p* < 0.01.

Increasing culture medium osmolarity by 100 or 200 mOsm/kg (i.e., +100, +200 mOsm), respectively, resulted in a quick nuclear translocation of transcription factor NFAT5, as qualitatively shown by representative snapshots of live confocal immunofluorescent imaging (Figure 1A). The nuclear translocation of NFAT5 is dependent on the quantity of the osmotic stress and is more pronounced under higher extracellular osmotic pressure. Within the physiological range (i.e., ≤ 500 mOsm/kg), this response appears to be almost linear (Figure 1B). The fold change in nuclear translocation further appears to be similar between pre-chondrogenic cells (ATDC5) and primary human chondrocytes (hAC) in vitro, upon signal normalization to histone H3 (Figure 1C). Increased osmolarity induced more NFAT5 nuclear translocation than the addition of FK506. Of note, increasing the extracellular osmolarity by +200 mOsm above plasma level (control) significantly inhibited ATDC5 proliferation (Figure 1D). Therefore, subsequently, only +100 mOsm/kg was studied as a relevant NFAT5-recruiting osmotic trigger, which would still optimally facilitate cell-based tissue engineering applications.

### 2.2. Calcineurin Inhibition by FK506 Does Not Affect Chondrocyte Proliferation at Physiological Osmolarity

**Figure 2:**
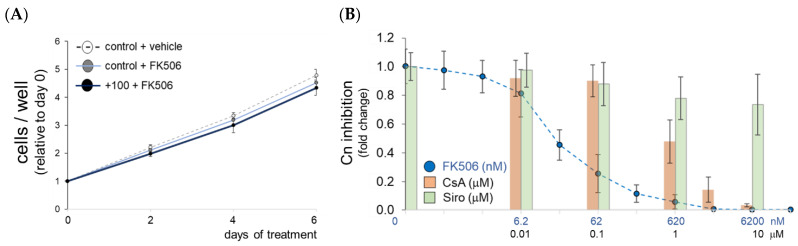
FK506 inhibits calcineurin activity, but not proliferation of ATDC5 cells. (**A**) Increasing the culture medium osmolarity by +100 mOsm/kg does not influence cell proliferation as compared to vehicle control (i.e., baseline plasma osmolarity), nor does FK506 (620 nM). (**B**) FK506 concentration more potently inhibits calcineurin (Cn) activity than cyclosporin (CsA). Dotted blue line: FK506 (nM); orange and green bars: CsA and Siro (μM), respectively. Data are means ± standard deviation of triplicate experiments.

The proliferation of ATDC5 cells at physiological culture osmolarity is not inhibited by the addition of FK506 (Figure 2A). Of note, FK506 is about 10 times more potent in inhibiting calcineurin activity in chondrocytes than cyclosporin A (CsA) (Figure 2B).

CsA served as an FKBP-independent, but potent, Cn inhibitor. Sirolimus (Siro, syn. Rapamycin), an mTOR inhibitor immunosuppressant, served as a Cn-independent negative control that is often used to show Cn-specificity of Cn-dependent drugs.

### 2.3. FK506 Stimulates Glycosaminoglycan Synthesis by Chondrocytes

**Figure 3:**
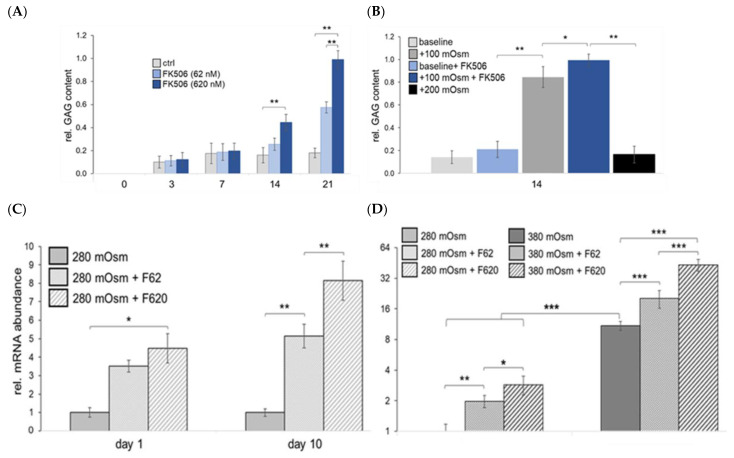
FK506 dose-dependently stimulates glycosaminoglycan and collagen synthesis in physosmotic ATDC5 cultures. (**A**) During chondrogenic differentiation, from day 14 on, FK506 significantly stimulates GAG production as compared to the control (ctrl) condition without addition of FK506. (**B**) Under physosmolarity (i.e., +100 mOsm), FK506 stimulates GAG synthesis. (**C**) Osmolarity- and FK506-dependent collagen type II expression in murine and (**D**) primary human chondrocytes. All data are means ± SD; *, F62 and F620 are 62 nM and 620 nM of FK506, respectively. Significant differences between groups are indicated as *, *p* < 0.05; **, *p* < 0.01; ***, *p* < 0.001.

In ATDC5 culture, from day 14 onwards, FK506 dose-dependently increased GAG content (Figure 3A). High-dose FK506 (620 nM) induced a two-fold increase in GAG content compared to the control and low-dose FK506 (62 nM) at day 14 (Figure 3A). The effect became more prominent at later stages of differentiation (day 21) when the highest dose of FK506 stimulated GAG synthesis about six times more than in the control (i.e., plasma level osmolarity). Interestingly, at day 14 of culture, the physiological osmolarity (+100 mOsm/kg) increased GAG content about fourfold in ATDC5 cells and FK506 further increased this difference as compared to the control (Figure 3B). Of note, increasing the extracellular osmolarity beyond +100 mOsm/kg decreased the overall GAG content of ATDC5 cultures (+200 mOsm/kg, black bar). We further studied *Col2a1* and *COL2A1* expression in ATDC5 and human chondrocytes (P1) to reveal an osmolarity- and FK506-depedency of the mRNA expression of this key ECM marker (Figure 3C,D). Of note, the fold change in FK506-depedent expression was most prominent in primary articular chondrocytes under physosmotic conditions (20- and 43-fold, respectively; Figure 3D). When cultured at plasma osmolarity, FK506 dose-dependently increased *Col2a1* mRNA abundance of the AT805-derived murine cell line by 3.5- and 4.5-fold, respectively, at early time points during differentiation (day 1) or 5.1- and 8.2-fold later during differentiation (day 10, Figure 3C).

### 2.4. Cartilage-Specific Physiological Osmolarity Improves ECM Composition

Aggrecan and collagen type II are the main ECM constituents of cartilage. Along with evaluating the effects of FK506 on the osmolarity-mediated GAG and collagen type II content, we also looked into the collagenous portion of the ECM of chondrocytes in more detail. Cartilage collagen fibrils consist mainly of collagen type II (to approx. 90% of the total collagen) and the quantitatively minor collagens IX and XI, as well as several non-collagenous fibril-associated proteins.

**Figure 4:**
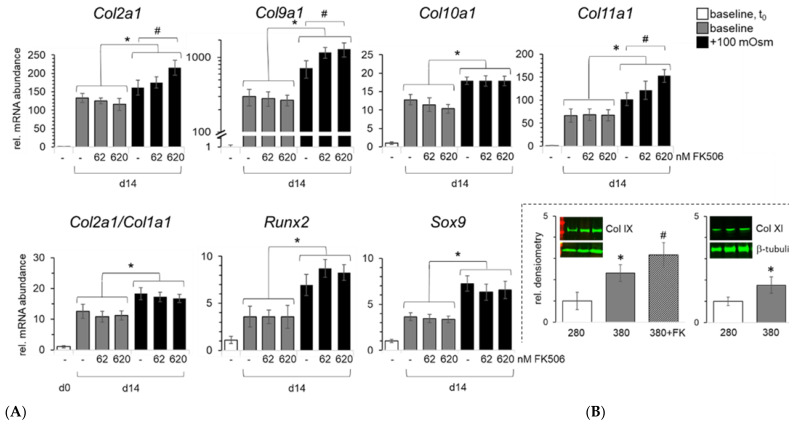
FK506 dose-dependently stimulates expression of fibrillar and minor collagens in physosmotic ATDC5 cultures. (**A**) Increased osmolarity enhances collagen mRNA expression, as well as key osteogenic (*Runx*2) and chondrogenic (*Sox*9) transcription factors, in ATDC5 cells. Addition of FK506 (62, 620 nM) dose-dependently increased mRNA abundance of *Col2a1*, *Col9a1* and *Col11a1* even further, but not that of *Col10a1*. Cells were cultured under plasma level osmolarity (i.e., baseline) and under physosmotic conditions (baseline +100 mOsm/kg; by addition of sterile sodium chloride from day 7 onwards). Shown are mRNA levels as quantified by RT-qPCR (n = 7), with a collagen type II vs. type I (i.e., Col2a1/Col1a1) expression ratio, next to expression of osteogenic (*Runx*2) and chondrogenic (*Sox*9) master regulators. Differences in osmolarity-related mRNA expression are indicated by * or by # for iso-osmotic FK506-specific differences, respectively. (**B**) Densitometrically determined differences in protein levels of collagen type IX (Col IX) and collagen type XI (Col XI), n = 3; β-tubulin (loading control); FK, 620 nM FK506; *p* < 0.05.

We previously looked into the osmolarity dependency of collagen type II expression, as it is the key marker of the proper chondrocytic phenotype [16,21]. During differentiation, its mRNA abundance increased about 130 times (d14 vs. d0, gray bars) in controls, while under physiological osmolarity at day 14 (black bars), *Col2a1* expression levels increased 160 times. *Col2a1* expression is further responsive to FK506 in a dose-dependent manner (62–620 nM), resulting in an additional 31% increase over iso-osmotic controls (Figure 4A).

We then studied the osmolarity and FK506 dependence of the expression of so-called minor fibrillar collagens, i.e., type IX and XI collagen, as well as those of collagen type X. All these collagens were expressed at a higher level under physosmotic conditions (Figure 4). Interestingly, similar to *Col2a1*, *Col9a1* and *Col11a1*, mRNA expression was upregulated by FK506 in a dose-dependent manner. Of these, *Col9a1* was relatively most prominently regulated. During differentiation (d14 vs. d0), *Col9a1* mRNA abundance increased about twice as much as that of *Col2a1* under control conditions. Physiological osmolarity doubled its mRNA expression and the highest dose of FK506 synergistically increased this by 50% as compared to the iso-osmotic condition (Figure 4A). Compared to *Col9a1*, the expression of *Col11a1* increased relatively less during differentiation (about 65-fold) and was not influenced by FK506 under control conditions. Under physosmotic conditions, *Col11a1* showed an FK506 dose-dependent increase in its expression (Figure 4A) with up to +50% (620 nM) of the iso-osmotic control. We confirmed that mRNA expression tends to be representative of the respective protein abundancies for collagen type IX and XI by immunoblotting (Figure 4B). In the case of collagen type IX, FK506 significantly improved collagen content in addition to the osmolarity-mediated effect. This is in line with our hypothesis that physosmotic culture and FK506 synergistically improve ECM maturation.

In contrast, *Col10a1* did not show an FK506 responsiveness (Figure 4A). While increasing osmolarity above physiological levels increased the ratio of collagen type II vs. collagen type I mRNA levels (*Col2a1*/*Col1a1*), FK506 had no additional effect. The expression of osteogenic key transcription factor *RUNX2* was also elevated by physosmotic conditions, but without clear regulation by FK506. Interestingly, the expression of the master regulator of chondrogenic differentiation *SOX9* was regulated in a similar way, but with a tendency towards dose-dependent suppression by FK506 (Figure 4A).

### 2.5. FK506 Improves Expression of Osmolarity-Mediated Cartilage-Specific ECM Key Markers

**Figure 5:**
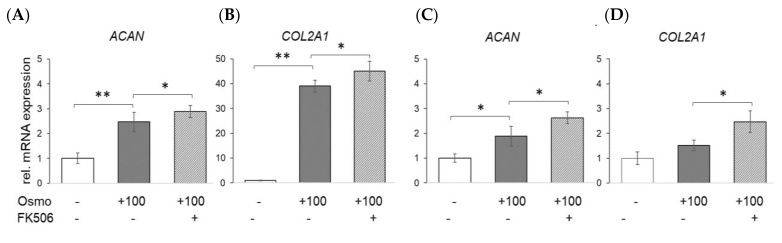
Effects of physosmotic culture and FK506 stimulation on human primary chondrocytes in vitro and in situ. (**A**,**B**) Chondrocytes were cultured for 7 days and (**C**,**D**) explants for 10 days, respectively. Relative changes in mRNA expression are shown as compared to iso-osmotic controls without FK506. Only the expression of main ECM markers aggrecan (*ACAN*) and collagen type II (*COL2A1*) are shown. Error bars indicate standard deviations from triplicate experiments; *, *p* < 0.05; **, *p* < 0.01.

As the large proteoglycan aggrecan and collagen type II are two of the main macromolecular key ECM constituents of articular cartilage, we further examined their expression in primary human chondrocytes (Figure 5A,B) and cartilage explants (Figure 5C,D), respectively. Considering a two-fold change in expression as the minimum for biological significance, physosmotic culture clearly induced *ACAN* and *COL2A1* in human chondrocytes. The general response to osmolarity and FK506 was less pronounced in explants as compared to isolated cells. However, FK506 significantly increased the expression of both key cartilage ECM markers in human chondrocytes in vitro and in situ.

### 2.6. Cartilage-Specific Physiological Osmolarity Improves Chondrocyte ECM Maturation

Based on the aforementioned results, we hypothesized that FK506 improved osmolarity-enhanced ECM maturation during chondrogenic differentiation. To test this, we investigated the expression of transforming growth factor-beta-induced protein (TGFBI), a TGF-β-inducible ECM protein that binds to, among others, type II collagen and seems essential during mesenchymal chondrogenesis [40]. Through controlling cell-collagen interactions, TGFBI has been shown to stimulate cell proliferation and differentiation, while inhibiting hypertrophy and mineralization [40].

**Figure 6:**
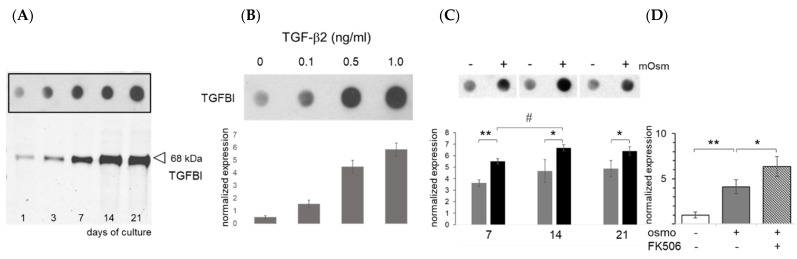
Expression of Transforming Growth Factor Beta Induced (TGFBI) protein. (**A**) Representative correlation of TGFBI expression during ATDC5 differentiation as dot blots and Western blotting (n = 2). (**B**) TGF-β-dependence of TGFBI protein expression at baseline and (**C**) its inducibility by osmolarity is shown (n = 3). (**D**) FK506-dependence of relative *TGFBI* mRNA expression (day 10, n = 5). −, plasma level osmolarity; +, +100 mOsm/kg; *, *p* < 0.05; **, *p* < 0.01; #, inter-day *p* < 0.05.

In ATDC5 cells, TGFBI is induced during chondrogenic differentiation (Figure 6A). Dot blotting showed TGFBI expression in ATDC5 cells was induced by physiological concentrations (0.1–0.5 ng/mL) of TGF-β2 (Figure 6B). Chondrocytes have been shown to release similar concentrations of TGF-β in response to an increased osmolarity (+100 mOsm/kg) [38]. At different stages of chondrogenic maturation, TGFBI was induced in ATDC5 cells under increased culture medium osmolarity (+100 mOsm/kg, Figure 6C). On day 10 of physosmotic culture, *TGFBI* mRNA expression upregulated 4.1-fold by conditions and an additional 1.5-fold by adding 620 nM FK506 (Figure 6D).

**Figure 7:**
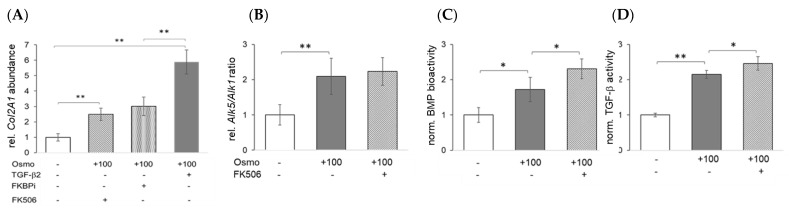
Modulation of collagen expression by FK506 is calcineurin and TGF-β dependent. (**A**) *Col2a1* mRNA expression (n = 5) and (**B**) Effect of osmolarity and FK506 on the mRNA expression ratio of *ALK5*/*ALK1* (n = 3), and on (**C**) BMP- and (**D**) TGF-β bioactivity, respectively, in chondrocytes (n = 3); *, *p* < 0.05; **, *p* < 0.01.

In line with earlier reports that increased osmolarity improves the secretion of TGF-β ligands in chondrocytes and chondrocytic cells from different species [38], physosmotic culture conditions (+100 mOsm/kg) alone increased *Col2a1* mRNA expression (Figure 7A). Adding powerful cytokines, such as TGF-β2 (1 ng/mL), increased *Col2a1* expression about six-fold (Figure 7A). Interestingly, under these conditions, FKBP12 inhibition (FKBPi, 1 μM) enhanced *Col2a1* expression to a similar extent as FK506 (Figure 7A), which suggests that FK506 may affect TGF-β signaling. While physosmotic culture also improved *ALK5* expression and thus shifted the *ALK5*/*ALK1* mRNA expression ratio (Figure 7B), FK506 did not significantly change this. However, physosmotic conditions alone already elevated BMP- and TGF-β bioactivity in chondrocytes, and FK506 was able to further enhance both (Figure 7C,D).

### 2.7. TGF-β Signaling Pathway-Dependency of FK506 Effects

FK506-mediated inhibition of calcineurin activity requires binding to its intracellular binding partner, FK506-binding protein-12 (FKBP12). Interestingly, FKBP12 is also known to bind and modulate TGF-β receptors in some cell types [41]. We showed earlier that Cn inhibitor FK506 further promotes the chondrocytic phenotype of human chondrocytes in vitro through stimulating TGF-β1 synthesis at plasma level osmolarity [9] and reported seemingly contradictory results when looking into TGF-β superfamily signaling upon osmotic stimulation [38]. To shed further light on how FK506 may induce collagenous ECM maturation in chondrocytes under physosmotic conditions, we next compared the effects of FK506 and cyclosporin A (CsA) as two FKBP-dependent and -independent Cn inhibitors, respectively, to those of the pharmacological inhibitors of TGF-β superfamily signaling and FKBP12.

**Figure 8:**
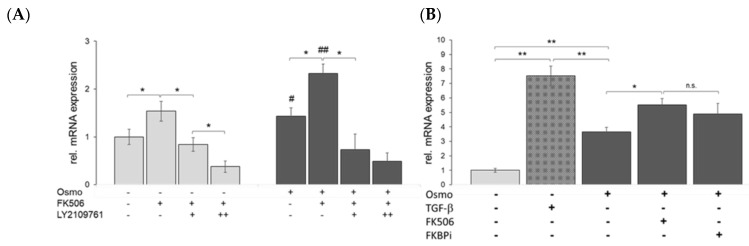

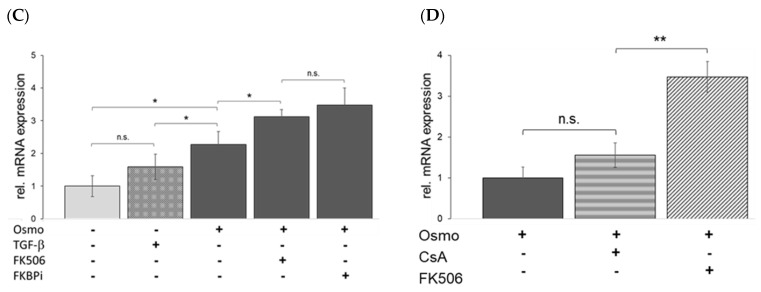
FK506 modulates TGF-β signaling in chondrocytic cells. (**A**) Effects of FK506 and TGF-βRI inhibitor (LY210961) on *SMAD3* expression. (**B**) Physosmotic *TGFBI* induction and modulation by TGF-β1, FK506, or FKBPi. (**C**) Physosmotic regulation of *Id1* expression and effects of TGF-β, FK506 and FKBPi (n = 5). (**D**) Effects of FKBP-independent and -dependent calcineurin inhibitors cyclosporin A (CsA, 1 μM) and FK506 (620 nM), respectively, on aggrecan expression (n = 3); osmo + = +100 mOsm/kg; LY210961 +, 0.1 μM; ++, 1 μM; isosmotic *, *p* < 0.5; **, *p* < 0.01; #, ## 380 vs. 280; n.s., not significant.

TGF-βRI inhibitor LY210961 decreased *SMAD3* mRNA abundances in a dose-dependent manner, while FK506 increased *SMAD3* expression in both control and physosmotic conditions (Figure 8A). FK506 was more effective at increasing *SMAD3* expression under higher osmotic values (i.e., plasma level control vs. +100 mOsm/kg). TGFBI showed a clear TGF-β ligand-dependent inducibility (7.5-fold), while physosmotic conditions increased TGFBI expression (3.6-fold, Figure 8B). FK506 and FKBP12 inhibition (FKBPi) showed an additional increase in TGFBI mRNA levels in physosmotic conditions (5.5- and 4.9-fold, respectively; Figure 8B). In contrast, Id1 expression increased 2.3-fold with increased culture medium osmolarity (+100 mOsm/kg), but was not affected by TGF-β (Figure 8C). Unexpectedly, FK506 and FKBP12 inhibition independently increased Id1 mRNA levels under physosmotic conditions (3.1- and 3.5-fold, respectively; Figure 8C). FK506 (FKBP12-dependent) upregulated the aggrecan mRNA level 3.47-fold, but CsA (FKBP12-independent) only increased the *Acan* mRNA 1.55-fold (Figure 8D).

## 3. Discussion

Earlier we showed that culturing chondrocytic and bone marrow-derived stromal stem cells from different species under increased osmolarity has a beneficial effect on chondrogenic phenotype [9,16,21] and that FK506 modulates the expression of several of these markers [17]. Recently, similar results have been reported for human adipose-derived mesenchymal stem cells (ADSCs) [42] too, which hints towards a broader applicability of this concept. Additionally, we reported that this involves TGF-β or BMP signaling under cartilage-specific physiological osmolarity [38].

In this study, we aimed at elucidating the mechanism by which FK506 modulates the physosmolarity-induced differentiation of chondrocytic (progenitor) cells and compared responses to physosmolar stimulation and FK506 between primary chondrocytes and chondrocytic cell lines. For the latter, ATDC5 cells were chosen as an optimized model for rapid and physiological matrix maturation [43,44]. Using the immunofluorescence imaging of NFAT5/NFAT5, we first showed a similar nuclear translocation between primary human chondrocytes and murine chondroprogenitor cells, confirming a similar response to elevated extracellular osmolarity (+100 mOsm/kg) in both cell types. Osmolarity was also used to differentiate ADSCs into nucleus pulposus cells [45], with potential epigenetic involvement, which matters in light of similar responses between cells from articular cartilage and nucleus pulposus [12]. However, details, such as osmotic baseline values, seem to influence the results and should be carefully considered, as we found that osmotic values in the range of about +100 mOsm/kg above plasma osmolarity do not negatively affect cell proliferation, while those around +200 mOsm/kg exert inhibiting effects. This is in agreement with reports by others [16,21,46,47], showing that culturing chondrocytic cells in a medium of 350–450 mOsm/kg appears to be optimal for tissue engineering and regenerative medicine, including closely related cell types, such as nucleus pulposus cells [12,13].

While calcineurin inhibition promotes the re-expression of chondrogenic markers in de-differentiated human chondrocytes in standard culture through the involvement of TGF-β signaling [9], the chondrogenesis of human synovial stromal cells was FK506- and SMAD signaling-dependent [48]. This is in agreement with FK506 improving the phenotype of primary osteoarthritic chondrocytes in vitro [16,17] and protecting the ECM of hyaline cartilage against collagen degradation in a rat model of OA in vivo [37]. Furthermore, in this paper from day 14 onwards during ATDC5 differentiation, an FK506-dependent accumulation of the non-collagenous ECM was shown using biochemical GAG quantification and proteoglycan (i.e., Acan) mRNA expression analyses. Interestingly, by raising the osmolarity by +200 mOsm/kg above plasma level, we observed a drop in GAG content. This is in line with earlier reports [21] and probably due to a reduced synthesis rate and paralleled by a compromised proliferation rate under these conditions.

Additionally, FK506 improved collagen type II expression not only in murine ATDC5 cells, but also in primary human chondrocytes to a similar extent. This is in line with other studies showing that FK506 can induce proteoglycan content in ATDC5 cells or collagen and aggrecan expression in human progenitor cells [17,49]. Our data further show that FK506, up to 620 nM, does not negatively affect the proliferation of chondrocytic cells under physiological osmolarity [30].

SOX9 (Sry-type high-mobility-group box 9) is expressed in all chondrocytes and regulated by osmolarity [21,50,51]; it is a key transcription factor essential for the expression of aggrecan [52] and most collagens [53]. As the main ECM constituents of cartilage, aggrecan and collagen type II, both benefit from physiologically osmotic culture conditions and the addition of FK506, our present data suggest that cartilage-specific physiological osmolarity improves the non-collagenous and collagenous composition of the ECM and thus likely influences its maturation. To this end, our data confirm earlier studies [17,37,49], but increasing the osmolarity to physiological levels also improved the collagen type II vs. collagen type I (*Col2a1*/*Col1a1*) expression ratio. FK506 did not significantly influence this further, but we speculate that upregulation of the osteogenic key transcription factor *RUNX2* by physosmotic conditions may be overruled by the higher co-stimulation of the dominant master regulator of chondrogenic differentiation, SOX9 [54].

As most collagenous ECM components are also controlled by Sox transcription factors [53], this may also explain why physosmolarity and FK506 also affect the expression of so-called minor collagens. Although cartilage collagen fibrils consist of approx. 90% of type II collagen, such minor collagens fulfill important stabilizing roles during the functional maturation of the ECM [24]. We selected collagen type XI as a minor member of the type II collagen-group of fibril-forming collagens, and collagen type IX as a member of the fibril-associated collagens with interrupted triple helices (FACITs), which do not form fibrils, but associate with fibril surfaces [55]. For instance, fibronectin (FN) interacts with type IX collagen and both co-localize in cartilage. Their interaction may be crucial for matrix integrity in vivo [56]. The importance of both collagens for a normal skeletal development are further underscored by the fact that transgenic collagen type II-deficient (*Col2a1*−/−) mice die at birth with a severely malformed skeleton and collagen type IX-deficient mice (*Col9a1*−/−) develop an early osteoarthritis-like phenotype in knee joints [57].

In situ, the FK506 treatment protected the collagenous network of articular cartilage in a rat model of osteoarthritis [37], in which chondrocytes already experience physiological osmolarities during joint movement [14,58]. Interestingly, a recent study has suggested that the expression of minor collagens may be compromised in osteoarthritic cartilage [59], which would be in agreement with the protective effects of FK506 in this earlier in vivo model. Of note, within the cartilage ECM, type IX collagen is covalently bound to the surface of type II collagen fibrils, and type IX collagen co-localizes there with FN, to improve matrix integrity in this tissue [56]. Our future studies will address the role of FN in assembling a functional cartilage matrix under these situations [60]. The importance of type IX collagen, which is also involved in a number of other ECM interactions, for ECM integrity becomes apparent from the fact that mechanical stress is required to cause joint cartilage degeneration in knockout mice [61]. In normal adult cartilage, collagen types IX and XI become more restricted to the pericellular matrix with increasing age [62] and type IX is concentrated pericellularly in skeletally mature cartilage, but more uniformly distributed in fetal tissue [63,64]. The predominantly pericellular localization of both type IX collagen and FN suggests that there may be a direct or indirect interaction with the chondrocyte itself, and a chondroprotective role for type IX collagen, in particular, has previously been postulated [65]. This has led us to hypothesize that type IX collagen is a molecular bridge between matrix components and the chondrocyte, and that FK506 stimulates ECM maturation by improving the expression of these minor collagens. Together, this underscores the therapeutic potency of FK506 in cartilage disorders.

We reported earlier that FK506 promotes chondrogenic marker expression in human chondrocytes through endogenous TGF-β production under standard culture conditions [9] and that physosmotic in vitro culture improves the expression of BMP and TGF-β isoforms and modulates TGF-β bioactivity [9,38]. Additionally, microarray data and in silico predictions hinted towards osmotic changes causing (post-) transcriptional TGF-β signaling responses in chondrocytes [39]. As TGF-β also mediates *Acan* expression in ATDC5 cells in a dose-dependent manner [66], it is not surprising that not only collagens, but also key proteoglycans are upregulated through *SOX9* by osmolarity. Interestingly, elevated osmolarity also activates several MAPKs [51,67], which have not been studied by us yet. However, the role of FK506 has remained elusive until now.

Therefore, we next investigated the expression of the TGF-β-induced protein TGFBI, an RGD-containing and type II collagen-binding ECM protein with an essential role during mesenchymal chondrogenesis [40]. TGFBI stimulates cell proliferation and differentiation through controlling cell-collagen interactions; its forced downregulation correlates with the upregulation of hypertrophic markers [40], and negatively affects the differentiation and mineralization of osteoblasts and hypertrophic chondrocytes [68]. This is in agreement with the idea that this molecule plays a key role in proper ECM maturation. We suspected this molecule to contribute to improving ECM integrity, as we earlier indicated that FK506 contributes to selectively suppressing catabolic markers in osteoarthritic human chondrocytes during in vitro expansion [17] and confirmed expression of this 68-kDa protein by immunoblotting techniques during chondrogenic differentiation of ATDC5 cells [4]. *TGFBI* mRNA expression was further induced by physosmotic conditions, and by FK506, which is interesting in light of the TGFBI knockout mice showing ECM degradation in articular cartilage [69]. Importantly, we showed that TGFBI is inducible by low-end physiological concentrations of TGF-β, similar to those reported to be released from chondrocytes upon increasing culture medium osmolarity by +100 mOsm/kg in vitro [38]. This provides new circumstantial evidence that physosmolarity, alone or in combination with FK506, may improve ECM maturation.

We next looked into the effects of osmolarity and FK506 on the expression ratio of the TGF-β superfamily type I receptors *ALK5* and *ALK1*, as well as into BMP- and TGF-β bioactivity in human chondrocytes under physosmotic conditions. The TGFβ family consists of over 30 members, including several mammalian prototypic TGF-β ligands and bone morphogenetic proteins (BMPs) [32].

There are seven type I-, five type II-, and some type III-receptors. Upon ligand binding to type II-receptors, type II- and type I-receptors dimerize and then activate the type I kinase function, which in turn phosphorylates R-SMADs (i.e., SMAD2/3 upon TGF-β binding or SMAD1/5/9 upon BMP-binding to TGFBRII/ALK5 or BMPRII/ALK1, respectively) [32]. Canonically, the ratio of Alk5/Alk1 is important because Alk5-mediated signaling increases SOX9, but decreases RUNX2, while Alk1-mediated signaling increases both *SOX9* and *RUNX2*. Alk1 thus favors terminal maturation, while Alk5 may prevent hypertrophy [30,70]. Elevating osmolarity increased the ALK5/ALK1 ratio, while FK506 had no effect. In contrast, BMP- and TGF-β specific bioactivities were both increased by FK506 under physosmotic conditions.

Second, proper SMAD signaling is essential to maintain a healthy cartilage ECM [32,71]. To reduce complexity, we further focused on SMAD3 expression, which has recently been shown to correlate well with its activation status in nucleus pulpous cells [72]. As compared to controls, physosmolarity alone increased SMAD3 expression, while FK506 surprisingly further improved SMAD3 expression under both conditions.

Of note, while the role of SMAD2 in cartilage biology is less clear, defective SMAD3 signaling is associated with hip and knee OA in humans, and SMAD3 KO mice suffer from rapidly progressive OA after birth. SMAD3 is believed to exert anti-hypertrophic effects by counteracting RUNX2 function and recruiting silencing histone class II deacetylases to RUNX2 responsive genes, such as *MMP13*, *COL10A1*, and alkaline phosphatase (*ALPL*) [32]. This is in agreement with our earlier finding that FK506 suppresses the same hypertrophic and catabolic genes in primary chondrocytes, while adding FK506 prevents this [17]. We then used LY210961, an ALK5/TGFBRII autophosphorylation inhibitor, to evaluate the contribution of this signaling route to osmotic-induced effects. LY210961 dose-dependently suppressed *SMAD3* expression, which is consistent with its positive feedback regulation [73]. ALK5 is commonly believed to activate SMAD2/3 to promote the expression of *PAI-1* or *TGFBI*, while ALK1 predominately activates SMAD1/5 to BMP- to specifically induce the expression of *Id1* [74,75,76], which is in agreement with our results, as *Id1* is not induced by TGF-β itself.

Interestingly, while *SMAD3* expression was dose-dependently suppressed by TGF-β type I receptor-specific inhibitors, *SMAD3* was upregulated in an osmolarity- and FK506-dependent manner. Hyperosmotic culture has recently been shown to have similar effects on cell types from other skeletal tissues, such as the nucleus pulposus cells from the intervertebral disk (IVD) [12,13]. Indeed, a recent report suggested that FK506 induces the TGF-β1/SMAD3 pathway independently of Cn inhibition to prevent IVD degeneration [77]. This is largely in agreement with our results showing that FK506, but not CsA, induces *Acan* expression. Thus, while Cn-NFAT signaling plays a vital role in bone remodeling, Cn activity does not seem to largely influence *Acan* expression under physosmotic conditions.

NFAT5, the key regulator of osmotic responses, is not controlled by Cn activity, as opposed to NFATc1–4 [78]. Rapamycin blocks FKBP12 binding to TGFBRI and reverses the inhibitory effect of FKBP12 on TGFBRI phosphorylation. However, further investigation is required.

Maintaining a favorable ALK5-to-ALK1 signaling balance in chondrocytic cells likely contributes to an overall stimulation of the collagenous and proteoglycan synthesis and ECM maturation, which potentially involves TGFBI. Both *Id1* and *TGFBI* are induced upon FKBP12 inhibition. FKBP12 can bind to, and modulate, TGF-β receptors in some cells [41], but also modulate BMP type I receptors in medulla cells [72]. We reported earlier that, in chondrocytes, blocking BMP signaling with dorsomorphin decreased *Col2a1* expression, independent of the osmolarity level [38].

However, developmental and stage-dependent differences contribute to variations in the overall response to osmolarity and FK506 between murine progenitor cells and adult human chondrocytes, or other cell types, in situ (i.e., in explants) or in vitro. Physosmolarity seems to stimulate the ALK1 signaling route leading to terminal differentiation [16,21], whereas FK506 appears to selectively activate the ALK5 signaling route.

It has been reported that FK506 competes with the GS domain for binding to FKBP12 and thereby dissociates FKBP12 from receptors, leading to an increase in wild-type receptor signaling by 2.5-fold [79], which fits to the fold change in our regulation. To this end, FKBP12 may provide a safeguard against leaky signaling from receptor multimerization [41]. Mutations in the binding sites of FKBP12 or TGFBRI abolished the interaction between these proteins, leading to receptor activation in the absence of added ligand. Thus, FKBP12 does not inhibit TGFBRI association with TGFBRII, but inhibits TGFBRI phosphorylation by TGFBRII. Notably, the involvement of FKBP12 may partially explain the seemingly contradictory findings of earlier studies, in which TGF-β RNAi under physosmotic conditions surprisingly increased *COL2A1* expression in primary human chondrocytes, while the pharmacological inhibition of BMP signaling decreased it in SW1353 cells [38].

In ATDC5 cells, a mutual regulation between BMP and TGF-β signaling pathways is established. TGF-β ligands are known to enhance BMP signaling, while BMP-2 can antagonize TGF-β signaling [80]. We also reported earlier that physosmotic conditions upregulated TGF-β ligands TGF-β2 and -β3 [38]. In chondrocytes, TGF-β2 more potently induces SMAD2 phosphorylation at low concentrations, such as those induced by physosmotic conditions, as compared to TGF-β1 and TGF-β3 [38,81], which adds further complexity. Using ATDC5 cells, it was further shown that ECM stiffness primes the TGF-β pathway to promote chondrocyte differentiation [82], which hints towards an additional physicochemical feedback loop. A limitation of our study is that we cannot exclude the indirect contribution of other factors resulting from increased osmolarity, such as macromolecular crowding [83,84].

Although we did not study the specific mechanism by which FK506 regulates TGF-β superfamily signaling in detail, we speculate that FK506 mainly regulates the TGF-β pathway through modulating FKBP12. We used TGF-β-specific targets (i.e., TGFBI) and BMP-specific target genes (i.e., Id1) to discriminate between the two different major TGF-β superfamily signaling pathways. We used FKBP-dependent (i.e., FK506) and FKBP-independent (i.e., CsA) Cn inhibitors [85] to discriminate between the different modes of action. However, we can only draw limited conclusions from the present study, as the crosstalk between TGF-β superfamily signaling routes is complex and involves positive and negative regulatory signaling loops and other SMAD proteins [86,87].

FK506 may alter the cytoskeleton of chondrocytic cells through increasing F-actin [88]. In turn, actin polymerization levels may regulate TGF-β receptor activation and signaling [89] to alter their cellular phenotype, which is currently under investigation.

Another limitation of our study is that we did not evaluate MAPK-signaling. It is known that TGF-β1-induced SMAD2/3 and SMAD1/5 phosphorylation are ALK5-kinase-dependent in primary chondrocytes and mediated by TAK1 kinase activity [32,70]. We also cannot exclude a contribution of other Cn-activated or -inhibited pathways.

In summary, we propose a novel circular route in which physosmotic culture improves TGF-β signaling to improve the maturation of the ECM, which in turn promotes terminal chondrocyte differentiation if FK506 does not shift the ALK5/ALK1 signaling towards the ALK5 route. In light of presently available data, the chondroprotective effects of FK506 appear conclusive. An improved understanding of how FK506 contributes to TGF-β superfamily signaling’s control of collagen expression under physiological culture conditions holds the potential to further improve the phenotypic stability of chondrocytes for future cell-based therapies of many other common orthopaedic conditions.

Future studies should investigate whether the effects of FK506 are cell type-specific or whether changes in FK506 concentration and/or timing during the chondrogenic differentiation of progenitor cells could achieve the same inhibiting effect on cell hypertrophy. This would make FK506 more useful for cartilage tissue engineering approaches using progenitor cells.

## 4. Materials and Methods

### 4.1. In Vitro Culture

Human primary chondrocytes were studied in vitro and in situ (i.e., as explants, using sterile disposable dermal 4 mm biopsy punches). Primary articular chondrocytes were isolated from cartilage explants with ethical approval (MEC 2004-322, MEC 08-4-028, MEC 2017-0183), or obtained from commercial sources (Cat.#CC-2550, Lonza group Ltd., Basel, Switzerland), and cultured as previously reported [16,90]. Osmolality of chondrocyte differentiation medium was determined using an Osmomat 030 (Berlin, Germany) and sterile sodium chloride stock solution (5 M) added to reach cartilage-specific physiological levels [16]. Cells were harvested at indicated timepoints for analyses of mRNA and protein expressions, as reported earlier by us [16,38].

Cell lines were cultured in monolayers using proliferation medium (DMEM/F12 (Invitrogen, Carlsbad, CA, USA), 5% FCS, 0.1% (*v*/*v*) gentamycin, 0.6% (*v*/*v*) fungizione and 1% NEAA (nonessential amino acids; all Invitrogen, Darmstadt, Germany), as previously reported [21]. Briefly, cells were plated, allowed to adhere overnight, and the following day chondrogenesis was initiated by changing the proliferation medium to differentiation medium. Differentiation medium comprised proliferation medium supplemented with 1% ITS (B&D Bioscience, Köln, Germany). Differentiation medium was changed every two days (day 0 to 10) and daily (from day 10 on). We showed earlier that at plasma osmolarity, ATDC5 cells acquire a chondrogenic phenotype from day 7 in differentiation with increased expressions of sex determining region Y box 9 (*SOX9*) and *Col2* [21]. Co-stimulation with indicated concentrations of CsA or Rapamycin occurred as described earlier for FK506 [17]. Cell proliferation was assessed by Trypan Blue (cat. T8154; Sigma, Hamburg, Germany) and DNA quantification as reported earlier [16]. Experiments were performed in replicates (n = 5).

### 4.2. Biochemical Analyses and Glycosaminoglycan (GAG) Content Determination

GAG content was determined using a Blyscan^TM^ Glycosaminoglycan Assay (Biocolor Ltd., County Antri, UK)) was used to determine sulfated proteoglycans and GAGs according to the manufacturer’s guidelines. The 1,9-dimethylmethylene blue chromophore (Amax. = 656 nm) was measured at 650 nm. Furthermore, mRNA abundance of the cartilage-specific proteoglycan core protein aggrecan (*Acan*) was determined by RT-qPCR (see Section 4.5 for details).

### 4.3. Immunofluorescent Analyses and Immunoblotting Techniques

To analyse direct effects of increased osmolarity on NFAT5 protein expression by IF, cells were cultured on 8-well culture slides (BD Falcon) and medium osmolarity changed by +100 or +200 mOsm/kg, respectively, for 30 min prior to imaging. Analyses of immunofluorescence were described earlier by us [91]. Briefly, an anti-NFAT5 antibody (kindly provided by Dr. Moo Kwon) with conditions as reported [92] and applied our established normalization procedure using a histone H3-specific antibody [93].

Western and dot blot analyses were essentially performed as reported elsewhere by us [17,21]. Routine Western blotting with chemiluminescent detection was recently reported by us [21] and used for TGFBI screening. Briefly, upon harvesting, cells were washed twice with PBS and lysed in RIPA buffer with addition of protease inhibitors (ThermoFisher Scientific). Total protein concentration was quantified by BCA assay according to the manufacturer’s protocol (Pierce, #23225) and subjected to 10% SDS-polyacrylamide gel electrophoresis (PAGE). Subsequently, electro-blotted nitrocellulose membranes (Protran BA83, Schleicher & Schuell) were blocked in 5% low-fat dry milk in 1X PBS, 0.05% *v*/*v* NP-40, incubated with the primary antibody 10188-1-AP, 1:1000; Proteintech, Manchester, UK) and detected with HRP-linked anti-rabbit IgG (CellSignaling, #7074; 1:2000) in combination with Pierce^TM^ ECL Western Blotting substrate (#32109, ThermoFisher Scientific, Waltham, MA, USA), according to the supplier’s instructions. Upon assuring antibody specificity, *dot blotting* was performed as described earlier by us [94] to screen TGFBI inducibility. Collagens (type IX and XI) were essentially blotted in the same way, but detected differently, using polyclonal collagen type IX and type XI antibodies (ABIN6260946, ABIN6257111; both 1:1500; antibodies-online, Aachen, Germany) and secondary goat anti-rabbit AlexaFluor800 antibodies (1:10,000; #A32735, Li-Cor Biosciences, Bad Homburg, Germany) as reported earlier [95]. All signals were quantified using the ImageJ.JS browser version at https://ij.imjoy.io/ (accessed on 4 April 2022).

### 4.4. Pharmacological Intervention and Calcineurin Activity Assay

Rapamycin (Sirolimus, #553210, Sigma-Aldrich, Zwijndrecht, The Netherlands), Cyclosporine (CsA, #30024, Sigma-Aldrich, Zwijndrecht, The Netherlands), and FK506 [37] were prepared as reported earlier [17]. Cells from monolayer cultures were washed twice with physiologic saline and lysed with Mammalian Protein Extraction Reagent buffer (NE-PER, Pierce, Bonn, Germany) according to the supplier’s instructions. Samples were stored at minus 80 °C until further use. Extracts were purified on a Micro Bio-Spin P-6 chromatography column (Bio-Rad Laboratories B.V., Veenendaal, The Netherlands) and protein concentrations were quantified using the BCA Protein Assay Kit (Pierce) in a microplate reader (VersaMax, Molecular Devices Ltd., Leiden, The Netherlands) [9]. Calcineurin activity was measured using the Calcineurin Cellular Assay Kit Plus (BioMol, Tebu-Bio, Heerhugowaard, The Netherlands) as described earlier [9]. Synthetic FKBP12 inhibitor ElteN378 (AOB17229; AOBious Inc., Gloucester, MA, USA) was dissolved in DMSO and subsequently diluted in culture medium (1:2000) to its effective concentration (1 μM) [96]. TGF-βRI blocker LY210961 was dissolved in DMSO (4 mM) and then diluted in medium to ≤ 10 μM; vehicle controls were omitted as DMSO concentrations < 0.1% never showed any adverse effects (data not shown).

### 4.5. Reporter Gene Assays and Gene Expression Analyses

Determination of BMP- and TGF-β bioactivity in SW1353 cells was essentially performed as recently described [38], using an established luciferase assay with a CAGA-luc SMAD2/3 reporter. Cells were seeded at 3 × 10^4^/well in 24-well plates and transiently transfected with 150 ng of the reporter construct and 75 ng of pRL-TK vector (Promega, Madison, WI, USA), an internal control for transfection efficiency, using FuGENE 6 transfection reagent (Roche Diagnostics, Basel, Switzerland). Twenty-four hours after transfection, cells were incubated for 2 h in medium containing 0.2% FCS, followed by 16 h incubation with 10 ng/mL TGF-β2 as a positive control, DMEM/F12 as a negative control or 300 μL conditioned medium. Twenty-four hours after stimulation, the firefly and Renilla luciferase activities were measured using the Dual-Luciferase Reporter Assay System (Promega, Madison, WI, USA).

Nucleic acid purification, quantification, cDNA synthesis and RT-qPCR are described elsewhere [13,14]. In line with basic MIQE guidelines for quantitative real-time PCR analyses [97], NanoDrop (ThermoFisher, Waltham, MA, USA) analyses derived RNA Integrity Numbers (RIN) of ≥ 8.5, being indicative of good template integrity. Samples with OD260/280 nm or OD260/230 nm ratios of the total RNAs outside 1.85–2.0 and 1.9–2.2, respectively, were excluded. Data were normalized to an index of three reference genes which were pre-evaluated to be stably expressed across samples. Relative expression was then calculated according to 2^−^^ΔCT^ method as reported earlier [94] and normalized to day 0, where applicable. Most qPCR assays were adopted from the literature; *Alk1*, *Alk5* [98], *COL2A1*/*Col2a1*, *Col1a1*, *Col10a1*, *SOX9*, *NFAT5*, *ACAN*/*Acan*, *RUNX2*, and an index of three reference genes (GAPDH, UBC, HPRT1) that were stably expressed across samples [21,94], *Col9a1*, *Col11a1* [99], *Id1* [94], and *TGFBI* [100]. For all primers E (%) = 93–97% was reported, justifying a 2^−^^ΔCT^ approach. *SMAD3* (NM_016769) specific primers were adopted from PrimerBank (ID31543222a1).

### 4.6. Statistics

Statistical analysis was performed using SPSS 20 (IBM, Armonk, NY, USA) or GraphPad Prism 6.0 (GraphPad Software, San Diego, CA, USA) and significance for ATDC5 experiments was determined by two-way ANOVA (with Bonferroni post hoc test) with a D’Agostino–Pearson omnibus normality test [21]. A univariate linear model analysis was used for pharmacological experiments, followed by a post-hoc Bonferroni test [17]. Statistically significant differences of *p* ≤ 0.05 are indicated.

## 5. Conclusions

Cartilage-specific physiological osmolarity increased the expression of key chondrogenic markers *SOX9*, *Acan*, and *Col2a1*. We identified a novel osmo-responsiveness of an RGD-containing, collagen-associated, and TGF-β-inducible protein TGFBI. To our knowledge, this is the first report of the upregulated expression of minor collagens *Col9a1* and *Col11a1* under physosmotic conditions. We postulate that this is indicative of ECM maturation in response to physosmolarity. Interestingly, FK506 appeared to improve chondrocytic marker expression in a rather calcineurin independent manner, by sequestering FKBP-12 and facilitating TGF-β superfamily signaling.

FK506 not only protects the collagenous ECM of articular cartilage, but may also improve its quality through stimulating the TGF-β superfamily signaling-mediated expression of important crosslinking components. While cell type-dependent differences require cautious interpretation, our results now better explain earlier seemingly contradictory findings and will potentially benefit future pharmacological interventions and cell-based cartilage repair strategies.

## Data Availability

Original data supporting findings of this study are available upon request through the corresponding author.
